# Human stem cell-based embryo models: from self-organization to recapitulating development

**DOI:** 10.3389/fcell.2026.1857249

**Published:** 2026-09-17

**Authors:** Dongsong Liu, Tianyao He

**Affiliations:** 1 State Key Laboratory of Reproductive Regulation and Breeding of Grassland Livestock, School of Life Sciences, Inner Mongolia University, Hohhot, China; 2 Key Laboratory of Animal Cellular and Genetics Engineering of Heilongjiang Province, College of Life Science, Northeast Agricultural University, Harbin, China

**Keywords:** bioprocess engineering, morphogen gradients, self-organization, stem cell bioengineering, stem cell-based embryo models

## Abstract

Human stem cell-based embryo models (hereafter referred to as embryo models) are a rapidly evolving field at the intersection of developmental biology and bioengineering. In this review, we examine design principles, material systems, and engineering approaches that enable the construction of models arising from pluripotent stem cells. We consider how three-dimensional environments, synthetic hydrogels, and spatiotemporal modulation of morphogenetic signals guide self-organization. Together, these factors enable the recapitulation of stages from blastocyst formation to early organogenesis. We then discuss opportunities of these engineered systems in disease modeling and developmental toxicity screening. We also address the scaling issues and biomanufacturing limitations that must be overcome to enable standardized, high-fidelity production.

## Highlights

- Stem cell-based embryo models establish a reproducible platform for studying human development by applying engineering principles to guide stem cell self-organization.
- Model fidelity requires precise biochemical process control of signaling pathways and engineered matrices that mimic the native microenvironment.
- Advanced biomanufacturing tools such as microfluidic reactors and intelligent biomaterials are essential for scaling production and enhancing structural precision.
- These models offer promising potential for developmental toxicity assessment and congenital disease phenotyping, decreasing dependence on animal-based experimental systems.

## 1 Introduction

Human early embryogenesis is a well-coordinated process of molecular regulation, cell division and differentiation, and morphological change. Humans advance from a single fertilized egg to the morula, blastocyst, bilaminar disc, and gastrula stages. This stage lays the foundation for subsequent fetal development. The major human organ systems, including the nervous and cardiovascular systems, begin to form during this stage. Due to practical and ethical limitations, these processes cannot be observed *in vivo*. Additionally, international guidelines prohibit the *in vitro* culture of human embryos for longer than 14 days after fertilization (Lovell-Badge et al., 2021). As a result, research on embryo implantation, gastrulation, and early organogenesis has relied largely on animal models (Rossant and Tam, 2018). However, these models often fail to fully capture specific human developmental processes, limiting their predictive value in key biomedical applications such as drug safety analysis, developmental toxicity assessment, and regenerative medicine. This understanding has accelerated the engineering of human embryo models that reliably replicate early developmental events. The current focus of work is to establish a robust and repeatable system to achieve higher structural fidelity of natural embryos through improved method control.

In recent years, the establishment of human embryo models has revolutionized in developmental biology (Zhu et al., 2023). By utilizing the self-organizing potential of pluripotent stem cells (PSCs) in defined culture conditions, these models recapitulate key stages of human embryogenesis. Representative models include blastoids, gastruloids, and post-implantation embryo-like assemblies (Shahbazi and Pasque, 2024). Although these models are lineage-deficient and cannot fully recapitulate *in vivo* embryogenesis, they offer several advantages over traditional models, including greater experimental tractability, scalability, and reduced ethical concerns. The emergence of embryo models has shifted experimental approaches from passive observation to active engineering of development that provides experimental access to stages of human development that were previously inaccessible (Rosner et al., 2025; Gantner et al., 2025; Li et al., 2026).

This review focuses on blastoids and gastruloids as the two most established classes of PSC-based embryo models that recapitulate development from pre-implantation to early organogenesis. While other models such as bilaminoids, SEM, and PASE are discussed, the primary emphasis is placed on blastoids and gastruloids due to their extensive characterization and widespread adoption in the field (Shahbazi and Pasque, 2024; Rosner et al., 2025).

This review summarizes recent advances in human embryo model research, with an emphasis on the bioengineering principles that guide their assembly. We first consider factors that underlie model formation: pluripotent stem cell states, common signaling pathways, and physicochemical principles of self-organization. We then discuss the methodology for promoting model formation, highlighting initial cell choice and engineered 3D culture environments. We also provide a systematic review of existing models, from pre-implantation blastoids to systems modeling gastrulation and early organogenesis. We place particular emphasis on how chemical engineering strategies (including biomimetic material design, microreactor culture, and process control) are enabling these models to transition from exploratory research tools into reliable and scalable production systems. Finally, we describe how embryo models are poised to transform developmental biology and underscore their increasing value in investigations of human embryogenesis, congenital diseases, and pregnancy failure.

## 2 The design blueprint of the embryo model: self-organization and signal network

### 2.1 Pluripotency spectrum: cellular origins of embryo models

The successful formation of mammalian embryo models is not accidental. It is the result of years of investigation into the fundamental biology that regulates natural embryogenesis. PSCs are classically defined by their capacity to differentiate into the three embryonic germ layers, namely, ectoderm, mesoderm, and endoderm, which give rise to all tissues of the organism (Evans and Kaufman, 1981; Martin, 1981). However, recent studies have shown that PSCs can also differentiate into extraembryonic lineages under certain culture conditions, particularly in naïve, expanded, and extended pluripotent states (Dong et al., 2020). This expanded potential has broadened the utility of PSCs for constructing embryo models that require both embryonic and extraembryonic components (Balubaid et al., 2024). The starting cell population is the first and most important factor determining the successful formation of embryo models. It should be derived from a developmentally appropriate stage and have the potential to differentiate into all embryonic and extraembryonic lineages. It is clear that PSCs exist in a continuum of distinct states. Therefore, the starting cellular state is one of the key determinants for successful formation of embryo models. It determines whether the model can successfully proceed through the correct sequence of development *in vitro* and form similar structures and patterns as those formed by natural embryos (Yanagida et al., 2021; Kagawa et al., 2022).

To illustrate these foundational cell lineages and their relationships, a schematic of early embryonic development and the origins of key stem cell types is provided (Figure 1). Current strategies to generate embryo models focus on using PSCs, trophoblast stem cells (TSCs), and extraembryonic endoderm (XEN) stem cells (Watts et al., 2018). These cells are usually derived from naïve or primed human PSCs or were reprogrammed (Savatier et al., 2017).

**FIGURE 1:**
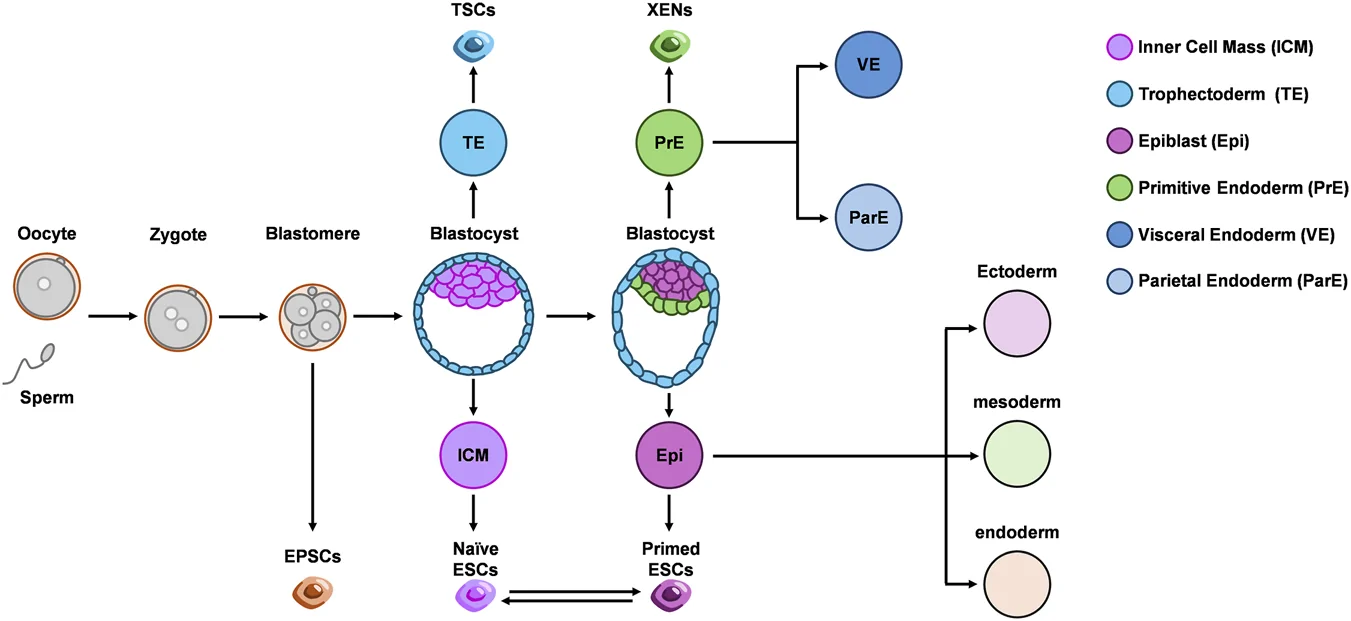
Schematic overview of early human embryonic development and the derivation of key stem cell types for embryo model construction. The flowchart follows a chronological order from top to bottom, starting from the zygote (fertilized egg) formed by sperm–oocyte fusion. After cleavage, the embryo develops into the morula, followed by the blastocyst, which comprises three distinct lineages: the trophectoderm (TE) (giving rise to the placenta), the inner cell mass (ICM) (forming the fetus), and the primitive endoderm (PrE). Upon implantation, the ICM further segregates into epiblast (Epi) and hypoblast, which generates the visceral endoderm (VE) and parietal endoderm (ParE). The epiblast then undergoes gastrulation to form the three germ layers. Key stem cell types used to model these stages are shown with their embryonic origins: naïve ESCs (derived from the pre-implantation ICM), primed ESCs (derived from post-implantation Epi), and extended pluripotent stem cells (EPSCs) (derived from early embryos with broader developmental potential). EPSCs differ from conventional ESCs in their ability to differentiate into both embryonic and extraembryonic lineages. Abbreviations: TE, trophectoderm; ICM, inner cell mass; PrE, primitive endoderm; VE, visceral endoderm; ParE, parietal endoderm; Epi, epiblast; ESC, embryonic stem cell; EPSC, extended pluripotent stem cell.

At the transcriptome level, naïve PSCs express high levels of KLF4, TFCP2L1, and KLF17, in addition to the core pluripotency factors OCT4 and NANOG (Wang et al., 2022). TSCs express CDX2, GATA3, TEAD4, and ELF5 while XEN-related cells typically express GATA4, GATA6, and SOX17 (Guo et al., 2024; Fan et al., 2021). These transcription factors control lineage specification and self-renewal. Functionally, naïve PSCs have broad developmental potential and can differentiate into epiblast (Epi), PrE, and trophoblast lineages (Massafret et al., 2024). In contrast, primed PSCs are generally restricted to differentiate into Epi only (Chen et al., 2025). TSCs differentiate into placental trophoblast cells and XEN stem cells give rise to yolk sac embryoid structures (Slamecka et al., 2024; Zylicz, 2020). In terms of structural modeling, co-culture of PSCs and TSCs generated blastoids that have morphology resembling pre-implantation blastocysts and exhibit preliminary lineage segregation (Wei et al., 2023).

Distinguishing naïve and primed pluripotency. Human PSCs exist along a spectrum, most notably the naïve and primed states. Naïve PSCs resemble the pre-implantation Epi; they can be derived from the ICM or obtained by reverting primed PSCs using small-molecule cocktails (e.g., 2i/LIF or t2iLGoY). They show broad plasticity, readily differentiating into Epi, PrE, and trophoblast, making them ideal for generating blastoids. Primed PSCs correspond to the post-implantation Epi; they are maintained with FGF2/Activin A and express OCT4/SOX2/NANOG but low KLF17/TFCP2L1. Their restricted capacity, which allows efficient formation of three germ layers but not extraembryonic lineages, makes them preferred for gastruloids and post-implantation models. Therefore, choosing the wrong pluripotent state for a given model type can drastically compromise structural fidelity and developmental progression, underscoring the need to match starting cell state to the intended developmental stage.

Complete embryoids contain three stem cell types: ESCs, TSCs, and XEN cells. These are also known as ETX embryos. By co-culturing these 3 cell types, researchers have generated ETX embryos that model post-implantation development, including gastrulation, amniotic cavity formation, and somite formation (Sozen et al., 2018). Under optimized conditions, PSC-derived models can also form amnion-like tissues, primordial germ cell-like cells (PGCLCs), and trilaminar germ layer structures (Kagawa et al., 2022; Liu L. et al., 2023). Under certain conditions, human PSC-derived embryo models can also form amnion, primordial germ cell-like cells, and trilaminar structures as well and can be useful models for investigating early human development.

Unlike conventional ESCs, which are generally restricted to embryonic lineages, extended pluripotent stem cells (EPSCs) exhibit enhanced developmental potency, enabling efficient differentiation into both embryonic and extraembryonic lineages. These cells have been captured from early cleaving embryos (2-cell to 4-cell stage) or reprogrammed using chemical cocktails (e.g., LCDM-based medium with LIF and WNT/HDAC inhibitors). This expanded potency makes them particularly well suited for constructing complete embryo models that include both fetal and placental components. Given this distinct feature, it is important to distinguish EPSCs from conventional ESCs. While both cell types express core pluripotency genes such as OCT4, SOX2, and NANOG, conventional ESCs (whether naïve or primed) are generally restricted to embryonic lineages (Epi), with limited capacity to form extraembryonic tissues such as TE or PrE. EPSCs, in contrast, are capable of differentiating into both embryonic (Epi) and extraembryonic (TE and PrE) lineages. Recently, several studies have reported that EPSCs can be used to form blastoids (Ávila-González et al., 2023). EPSCs have strong self-renewal ability and high developmental potential, expressed core pluripotency genes such as OCT4, SOX2, and NANOG (Yoshimatsu et al., 2023). When EGF induction, EPSCs can effectively activate transcriptional network of trophoblast lineage, including CDX2, GATA3, TEAD4 (Fan et al., 2022). The expression of these transcription factors improves the differentiation potential of EPSCs toward TE lineages. With this, EPSCs can give rise to multiple trophoblast subtypes, including cytotrophoblasts and syncytiotrophoblasts. EPSC-derived blastoids display an anatomy similar to that of natural human blastocysts (DiStefano et al., 2018). Their transcriptome profiles are largely comparable to those of pre-gastrulation human embryos. These findings suggest that EPSC-derived blastoids can serve as a useful system for modeling early human development.

In summary, the pluripotency state of starting cells sets the stage for constructing embryo models and also determines their subsequent developmental direction. Naïve pluripotency is the foundation for producing the most faithful blastocyst-like structures, and the primed state is the preferred starting point for gastrulation and early organogenesis. Having thus established what the preferred starting cell is, we next ask how these cells are instructed to organize into structured embryos, and discuss the signaling pathways that control cell fate determination.

### 2.2 Signaling pathways governing cell fate determination

The self-organization of PSCs into embryo-like structures is controlled by evolutionarily conserved signaling pathways that coordinately direct cell fate decisions, spatial patterning, and morphogenesis. To faithfully recapitulate early mammalian development, these *in vitro* models depend on the spatiotemporal integration of a core signaling network that includes BMP, WNT, NODAL, FGF/ERK, and Hippo pathways (Wang et al., 2025). The spatiotemporal relationships among these pathways are illustrated in Figure 2. These signaling pathways are biochemical toolboxes that can be used to program cell fate. Consequently, the fidelity of embryo models depends on accurately mimicking dynamic interactions, concentration gradients, and timings of pathway interactions that steer development from initial lineage specification to advanced axial organization.

**FIGURE 2:**
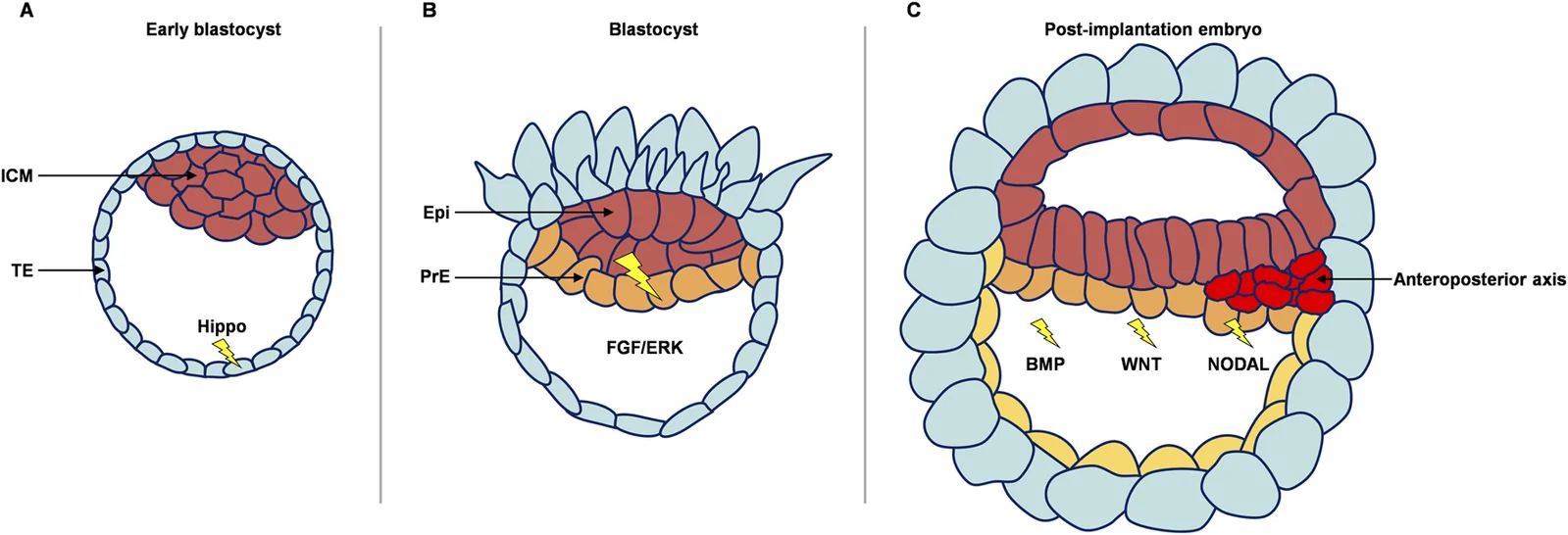
Spatiotemporal coordination of core signaling pathways during early human embryo development. This schematic illustrates the major signaling pathways that regulate cell fate decisions from the pre-implantation to the post-implantation stages. **(A)** The pre-implantation stage is depicted, during which the blastocyst forms and the first two lineage segregation events occur. Hippo signaling spatially distinguishes the outer trophectoderm (TE) from the inner cell mass (ICM). **(B)** FGF/ERK signaling drives the segregation of the ICM into the epiblast (Epi) and primitive endoderm (PrE). **(C)** The post-implantation stage is depicted, during which epiblast cells undergo gastrulation. A spatiotemporal signaling cascade involving BMP, WNT, and NODAL establishes the posterior-anterior axis and induces germ layer formation. Color code: pink/red, embryonic or outer cell lineages; blue, extraembryonic cell lineages; orange/yellow, inner cell lineages. Lightning bolts indicate active signaling events. Abbreviations: TE, trophectoderm; ICM, inner cell mass; Epi, epiblast; PrE, primitive endoderm.

Within this network, these pathways form a hierarchy and display complementary relationships. BMP signaling acts as an initial driver of extra-embryonic lineages. BMP signaling is usually activated at the periphery and drives TE and amniotic fates by expressing key markers such as CDX2 (Bernardo et al., 2011). Treatment with the BMP inhibitor LDN193189 reduces CDX2 expression and impairs TE formation, thereby compromising normal blastocoel development in blastoids (Graham et al., 2014). Then, WNT signaling acts as an initial inducer of mesoderm and an axial organizer. For example, pharmacological activation of WNT signaling with WNT agonists such as CHIR99021 induces BRA/TBXT expression and establishes a posterior-to-anterior activity gradient of WNT activation, triggering subsequent axial elongation (Beccari et al., 2018). Inhibition of WNT signaling by IWP2 abolishes BRA/TBXT expression and disrupts anterior-posterior axis establishment, preventing gastruloids from forming elongated tail-like structures (Bergmann et al., 2022; Marikawa, 2022). The NODAL pathway then potentiates mesendoderm specification and works in conjunctions with WNT to define anterior-posterior axis (Kunwar et al., 2003). Inhibition of NODAL/Activin signaling by SB431542 blocks mesendoderm specification and impairs axial elongation, consistent with the essential role of this pathway in anterior-posterior axis establishment (Chhabra et al., 2019).

Parallel to these axial patterning events, the FGF/ERK and Hippo pathways have essential functions at the very earliest stages of cell fate specification. The FGF/ERK cascade is required to pattern the ICM of the blastocyst. Specifically, the FGF4 ligand predominantly signals through the FGFR2 receptor to activate the MAPK1/2 cascade ERK. Activation of this cascade drives PrE specification in a non-cell-autonomous manner (Kang et al., 2017; De Caluwé et al., 2019). It also drives Epi cells to exit the naïve pluripotent state and prepare for subsequent differentiation (Kang et al., 2017; De Caluwé et al., 2019). Pharmacological inhibition of FGF/ERK signaling by PD0325901 eliminates GATA6 and SOX17 positive PrE cells (Schrode et al., 2014; Van der Jeught et al., 2013). As a result, all ICM cells adopt a NANOG positive Epi fate. These findings confirm that FGF/ERK signaling is required for PrE specification. Biochemical signals are complemented by the Hippo pathway, which translates cell position into distinct lineage fates. In outer cells, unphosphorylated YAP localizes to the nucleus and interacts with the transcription factor TEAD4 to drive a TE genetic program. In contrast, in inner cells, LATS1/2 kinases in the Hippo pathway phosphorylate YAP to retain it in the cytoplasm and thereby suppress the TE program and ensure proper segregation of the TE from the ICM (De Caluwé et al., 2019; Nishioka et al., 2009). Knockout of YAP, a core effector of the Hippo pathway, leads to loss of CDX2 and GATA3 expression in outer cells and prevents TE formation (Nishioka et al., 2009). In contrast, constitutive activation of YAP causes inner cells to aberrantly express TE markers and disrupts proper ICM specification.

Importantly, these signaling pathways do not function in isolation. Instead, they are embedded in an integrated network with a temporal activation cascade (BMP WNT NODAL) and complementary spatial gradients, such as anterior BMP versus posterior WNT/NODAL (Chhabra et al., 2019). This network is further interconnected with the FGF/ERK and Hippo pathways. Functional evidence from using specific small-molecule inhibitors of these pathways (e.g., LDN193189 for BMP, IWP2 for WNT, and SB431542 for NODAL/Activin) demonstrates that the proper function of this entire network is required for robust generation of complex morphological features *in vitro*, including tailbud-like structures expressing genes involved in somitogenesis and cardiac mesoderm precursors (Neupane et al., 2025). Hence, embryo models provide an excellent system for deconstructing signaling center-guided self-organization events that are impossible to access during post-implantation human development (Pedroza et al., 2023).

In summary, the coordinated actions of these pathways provide spatiotemporally ordered biochemical cues that guide cell populations. However, translating these chemical cues into morphological structures and morphogenetic forces depends on deeper self-organizing principles, as discussed below.

### 2.3 Physical and chemical principles of self-organization

The formation of embryo models depends on the self-organizing capability of stem cells, which operates according to physical, chemical, and biological principles. Cell sorting, pattern formation, and mechanoregulation are involved. Intercellular connections, uneven distribution of signals, and migration of the cytoskeleton are all physical and chemical principles that drive the spatial sequencing of cells.

According to the Differential Adhesion Hypothesis, cells self-sort in co-culture by differentially expressing adhesion molecules such as E-cadherin. These molecules modulate intercellular adhesion strength and tissue surface tension, driving phase separation-like behavior (Nicol and Garrod, 1979; Steinberg, 2007). During mouse embryogenesis, E-cadherin-mediated adhesion switches on at compaction at the 8-cell stage (Fleming et al., 2001). E-cadherin enriches at basolateral membranes, driving blastomere polarization and proto-epithelial phenotypic state. This adhesion pattern then guides cell division orientation and spatial segregation: daughter cells that fail to retain apical components localize externally and differentiate into the TE, while ICM-like cells become internalized (Stephenson et al., 2012). Importantly, inhibiting E-cadherin function blocks this segregation, causing ICM-like cells to be mislocalized into the TE lineage (Bedzhov and Zernicka-Goetz, 2014). Subsequent studies have shown that tissue surface tension is not governed by adhesion binding energy but rather by cortical tension, specifically actomyosin contractility at cell-cell interfaces (Méhes et al., 2023). Thus, cell sorting should be a self-organizing process in which cells balance adhesion and cortical contractility to minimize interfacial tension, and thereby achieve the precise spatial architecture necessary for development.

In addition to signaling cascade activation, a more important role in spatial patterning is played by a self-organizing mechanism known as “local self-activation and long-range inhibition” (Caldarelli et al., 2024). Stable concentration gradients and repetitive patterns of morphogens like BMP, WNT and NODAL are formed in cell populations through the synergy of local self-enhancement and long-range inhibition (Bedekar et al., 2021). This local enhancement-long range inhibition mechanism generates periodic spatial patterns in cell populations. For example, in the posterior region of the embryo, WNT signaling activates its own expression and promotes inhibitory factors (ten Berge et al., 2008). This creates a self-organizing activity gradient along the anterior-posterior axis through long-range inhibition, which guides primitive streak formation and axial extension. BMP signaling uses a similar activation-inhibition logic to set expression boundaries in peripheral regions and forms complementary patterns with inner signals (E et al., 2016). This local activation–long-range inhibition mechanism converts discrete biochemical signals into continuous spatial patterns and provides an intrinsic dynamic basis for the self-organization of embryo-like structures from homogeneous cell aggregates.

Cell fate is determined not only by biochemical signals but also directly by mechanical force, a process termed mechanotransduction (Alasaadi and Mayor, 2024). The Hippo pathway is a typical example. Its core effector YAP/TAZ can directly sense mechanical stimuli (Nardone et al., 2017; Driscoll et al., 2015). When outer cells are stretched, actomyosin contractility is enhanced. This leads to more frequent nuclear localization of unphosphorylated YAP, where it cooperates with TEAD4 to activate trophectoderm-specific genes (Nishioka et al., 2009). When inner cells are spatially confined, they experience compression and decreased cytoskeletal tension, and then the Hippo kinase cascade is activated to phosphorylate YAP and retain it in the cytoplasm, thereby suppressing the expression of its target genes (Alasaadi and Mayor, 2024). This mechanosensing process directly converts macroscopic physical constraints into biochemical signals that determine cell differentiation, thus ensuring spatially organized cell fate patterning.

Taken together, these three self-organization principles operate in a coordinated manner. Differential adhesion first drives cells to separate into distinct positions. This spatial segregation provides positional information. Cells then use this information to convert physical forces into biochemical signals through the Hippo-YAP pathway. Concurrently, reaction-diffusion mechanisms, driven by local activation and long-range inhibition, amplify and stabilize these spatial patterns to guide robust morphogenesis. The interplay of these principles enables stem cells to recapitulate embryonic architecture *in vitro*. The coordinated interplay of these three principles is summarized schematically in Figure 3.

**FIGURE 3:**
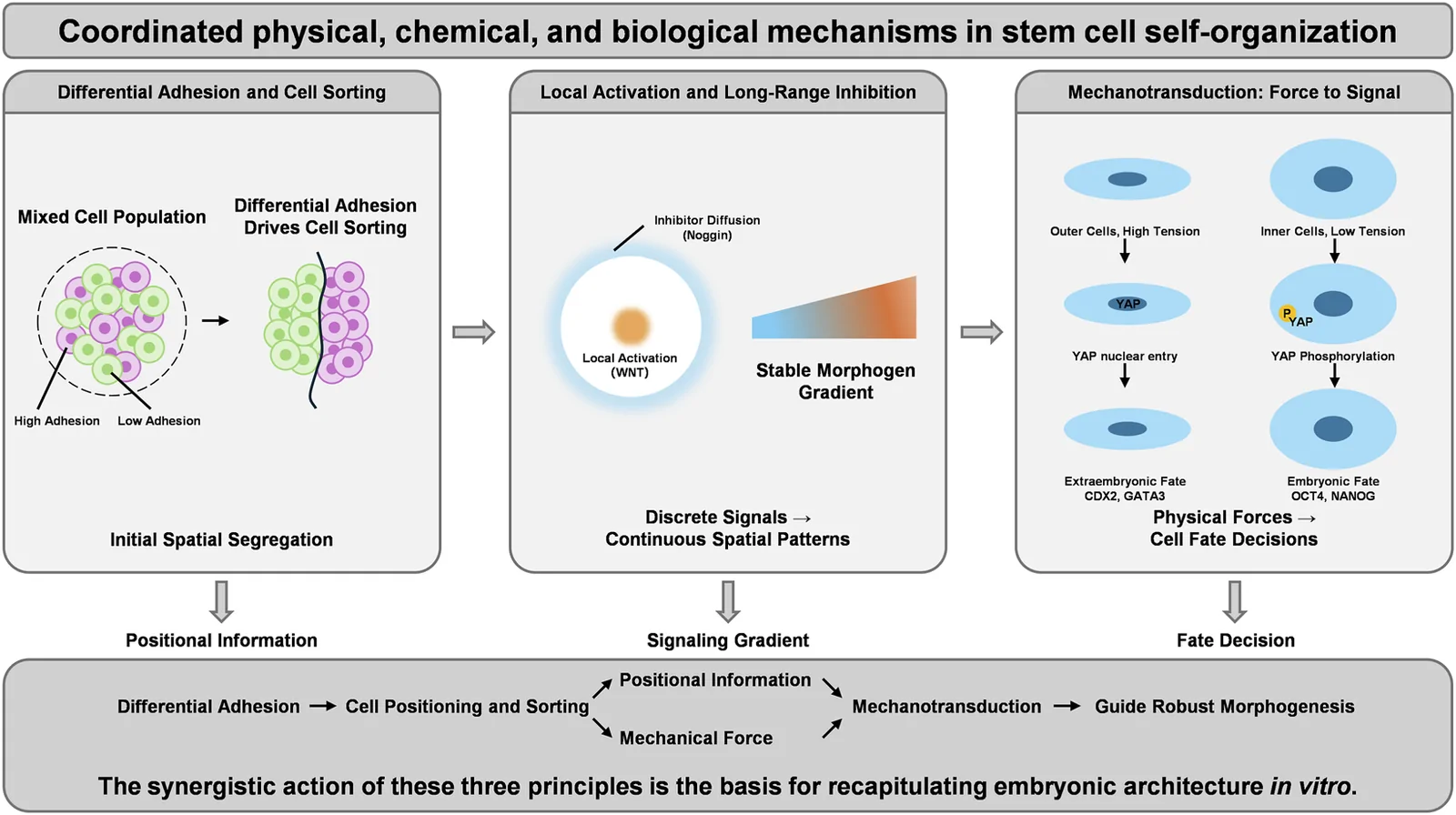
Integration of the three core physical and chemical principles governing embryo self-organization. The schematic illustrates how differential adhesion, reaction-diffusion (local activation and long-range inhibition), and mechanotransduction coordinate to guide embryo model formation (Left) Differential adhesion, mediated by E-cadherin, drives cell sorting and establishes initial spatial segregation (Middle) The reaction-diffusion mechanism, through local self-enhancement and long-range inhibition, converts discrete signals into stable morphogen gradients that pattern cell populations (Right) Mechanotransduction translates mechanical forces (stretch vs. compression) into biochemical signals via the Hippo-YAP pathway, leading to lineage-specific gene expression (TE genes vs. pluripotency) (Bottom) The three principles operate synergistically: differential adhesion provides positional information, mechanotransduction converts forces into signals, and reaction-diffusion amplifies and stabilizes spatial patterns to guide robust morphogenesis.

## 3 Technical methodologies guiding embryo model construction

The construction of embryo models is an engineered assembly *in vitro* of the cellular origins, signaling pathway regulation, and physicochemical principles of self-organization observed *in vivo* with those of native embryos. Two technical pillars are essential for constructing embryo models: selecting and preparing starting cells with appropriate developmental potential, and designing three-dimensional microenvironments that guide self-organization through spatiotemporal control of signaling cues. In this section, we condense relevant elements of published culture protocols to extract methodological insights that we hope will aid in producing embryo models with higher fidelity to *in vivo* biology.

### 3.1 Selection and preparation of starting cell types

One of the first decisions when building embryo models is the choice of starting cell population. The ability to self-organize into embryo-like models, and the degree to which natural embryonic architecture can be recapitulated, depend on whether we have access to developmentally competent starting cells.

For modeling human embryogenesis, there is a clear choice between hESCs and human induced pluripotent stem cells (hiPSCs) (Figure 4). hESCs are derived directly from blastocysts and have a native epigenetic state and stable developmental potential (Pastor et al., 2016). This makes them the natural cell source when the goal is to model normal early human development. When induced to form blastocyst-like structures (blastoids), hESCs generate the most faithful models and best recapitulate early embryogenesis. On the other hand, hiPSCs are reprogrammed from patient somatic cells such as fibroblasts and allow the modelling of patient-specific diseases (Liu et al., 2021). Powerful tools to study genetic forms of early developmental disorders have been recently achieved by deriving blastoid-like structures (iBlastoids) from hiPSCs (Liu et al., 2021) (see also (Luijkx et al., 2026) for a recent benchmarking review). However, these iBlastoids have limitations. They may retain epigenetic features inherited from the original somatic cells, and their developmental potential is often restricted (Pastor et al., 2016). In summary, the cell source choice depends on our research goals. While hESCs are optimal for the identification of universal developmental mechanisms, hiPSCs are an indispensable choice for disease modelling and personalised applications.

**FIGURE 4:**
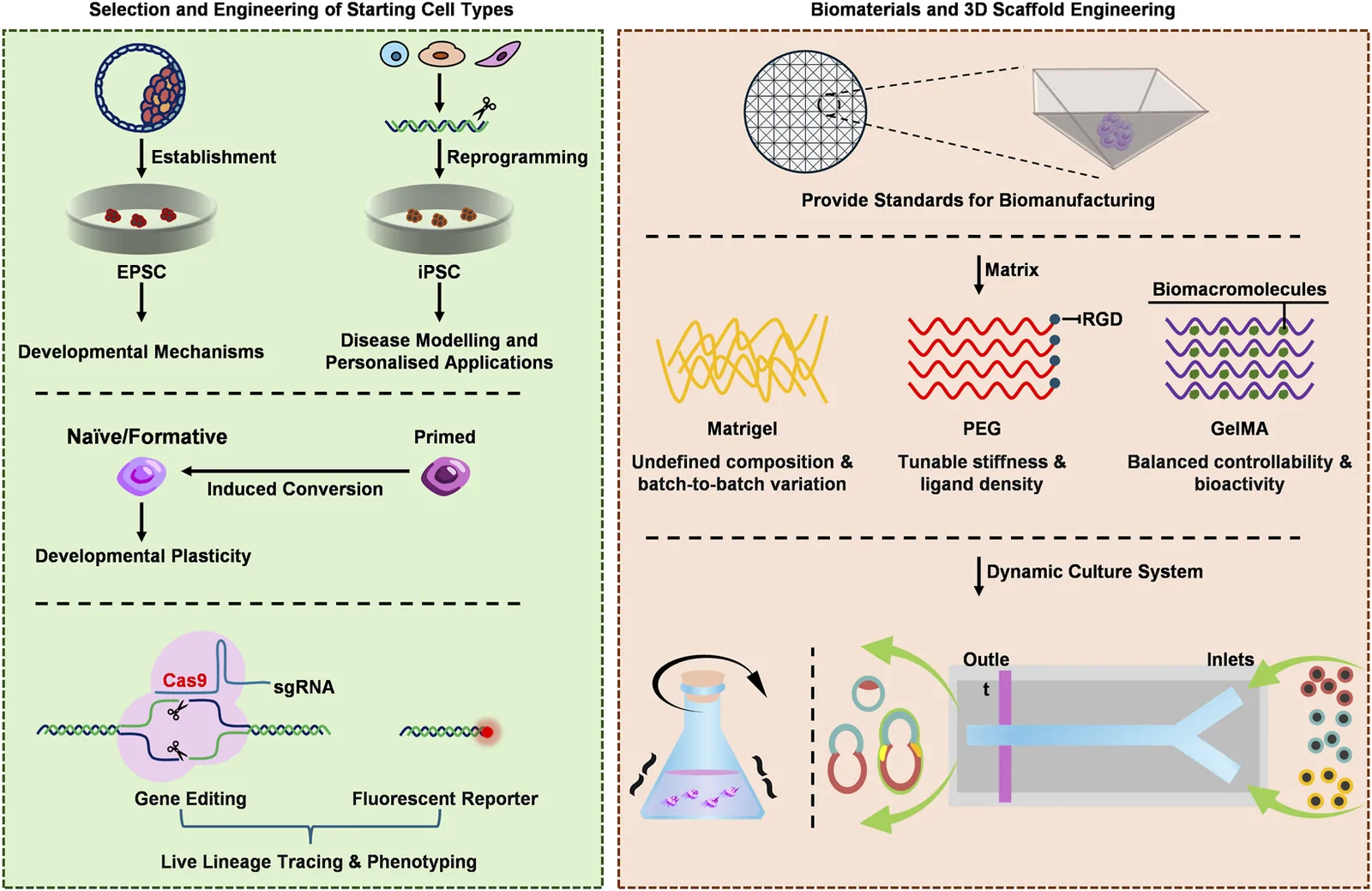
Engineering the cellular and physical microenvironment for embryo model construction. This schematic illustrates the two core engineering pillars for embryo model assembly. Left: selection of starting cells—hESCs derived from blastocysts or hiPSCs reprogrammed from somatic cells—with EPSCs offering extended lineage potential. Modulation of pluripotency states using small-molecule cocktails (primed ↔ naïve/formative conversion), enhancing developmental plasticity. Right: three-dimensional culture systems incorporating microwells (standardizing aggregate formation), defined synthetic hydrogels (PEG, GelMA) with tunable physicochemical properties, and dynamic bioreactors (perfusion) for improved nutrient transport. Gene editing and fluorescent reporters enable live lineage tracing and disease modeling. Abbreviations: hESC, human embryonic stem cell; hiPSC, human induced pluripotent stem cell; EPSC, extended pluripotent stem cell; PEG, polyethylene glycol; GelMA, gelatin methacryloyl.

Regardless of the cell source chosen, subsequent fine-tuning of its pluripotency state is required. This state modulation applies to both hESCs and hiPSCs, and will be discussed separately below. This is achieved through specific small molecule combinations that regulate signaling pathways, enabling transitions between primed, naïve, or formative pluripotency states. For instance, conventional primed hESCs need to be reset to a naïve state (closer to the pre-implantation embryo) for efficient blastocyst-like structures formation. This process can be facilitated by the 2i/LIF system (including PD0325901, a MEK inhibitor, and CHIR99021, a GSK3β inhibitor) or by other naïve culture conditions such as PXGL, 2iL + Go, and 5iLA (Guo et al., 2016; Takashima et al., 2014; Theunissen et al., 2014). Another condition, SILAF (consisting of 2i, LIF, ascorbic acid, and forskolin), has also been used for naïve cell maintenance (Duggal et al., 2015). However, unlike PXGL, 2iL + Go, and 5iLA, SILAF-cultured cells have not yet been reported to generate blastoids (Guo et al., 2024). Naïve-state cells or intermediate states during the state switching process display higher developmental plasticity and can differentiate into Epi, PrE, and TE lineages (Guo et al., 2024). Therefore, these cells can self-organize into more embryo-like structures with higher morphological similarity to the natural blastocyst, and have better reproducibility and fidelity in lineage composition and transcriptome level. It is worth noting that the conversion from primed to naïve pluripotency is not limited to hESCs. HiPSCs can also be reprogrammed to a naïve state using similar small-molecule cocktails, such as the 2i/LIF system or the more optimized PXGL culture condition (PD0325901, XAV939, Gö6983, and hLIF). However, the efficiency of naïve conversion and the stability of the resultant naïve state can vary depending on the somatic origin of the hiPSCs and their epigenetic memory. These iBlastoids can exhibit transcriptional features resembling those of natural human blastocysts (Balubaid et al., 2024; Liu et al., 2021). In parallel, hESC-derived blastoids have been established from naïve PSCs with high efficiency and faithful lineage composition (Kagawa et al., 2022; Guo et al., 2024). Collectively, these findings suggest that both hiPSC- and hESC-based systems can model early human development, although systematic side-by-side comparisons of their efficiency and fidelity remain limited. Nevertheless, careful quality control is essential to ensure faithful conversion. This includes checking pluripotency marker expression (e.g., KLF17, TFCP2L1), mitochondrial morphology, and X-chromosome reactivation status in female lines. When using naïve hiPSCs for embryo model construction, researchers should note that residual epigenetic memory from the original somatic cells may occasionally bias differentiation toward certain lineages. However, current evidence suggests that fully converted naïve hiPSCs perform similarly to naïve hESCs in most blastoid and gastruloid assays. Only after passing these quality control criteria can the prepared cells be used for subsequent genetic engineering and model construction.

Once the optimal starting cells are selected and prepared, genetic engineering tools can further validate and enhance their utility (Khampang et al., 2023; Lodewijk et al., 2025). Lineage reporting systems are established by expressing fluorescent reporter genes in target lineage-specifying genes (e.g., SOX2, TBXT, SOX17), which can be non-invasively visualized and dynamically tracked during the differentiation process (Zhang et al., 2017). This allows researchers to monitor how prepared cells behave during embryoid formation, for instance, tracking how Epi induces maturation of polar TE. In addition, CRISPR-Cas9 technology directly establishes genotype–phenotype relationships in human model systems. For example, YAP1 knockout or expressing its constitutively active form can reveal the core functions of signaling pathways in lineage specification, blastocoel formation, and implantation. By combining live imaging, single-cell omics, and genetic perturbation, researchers can integrate multi-scale information ranging from molecular mechanisms to overall morphogenesis (Xiao et al., 2022).

To implement the lineage reporting systems mentioned above, reporter knock-in should be introduced at the pluripotent stem cell stage before embryoid aggregation. For example, SOX2-GFP can be used for Epi, TBXT-mCherry for mesoderm, SOX17-tdTomato for PrE, and CDX2-mNeonGreen for TE. Introducing reporters at this stage ensures uniform labeling and uninterrupted tracking of all descendant cells (Vanslam et al., 2019). Introducing reporters after aggregation risks mosaic expression and incomplete lineage coverage.

### 3.2 Biomaterials and 3D scaffold engineering

The development of embryo models has progressed from conventional 3D cultures with spontaneous self-organization to engineered assembly systems (Figure 4). Here, we focus on physical scaffolds such as AggreWell microwells. These devices rigorously control the size and consistency of initial cell aggregates, thereby reducing the stochasticity and asynchrony that often complicate embryo model formation (Chen et al., 2023). Microwells serve as microscale bioreactors that provide standards for biomanufacturing (Iworima et al., 2023). This physical confinement, achieved by seeding about 30 naïve human pluripotent stem cells (hPSCs) per microwell, significantly enhances the efficiency of embryoid formation. By restricting cell spreading and directing lumen formation to low-tension regions, microwell confinement promotes symmetry breaking, a critical event that initiates spatial patterning and lineage segregation in embryo models (Girgin et al., 2021). That is, physical confinement thus provides a starting point for establishing a physical platform that can standardize and scale up embryo model manufacturing.

When it comes to the construction of embryo-like models, engineered materials serving as alternatives to the extracellular matrix are essential to direct the self-organization process. Matrigel is widely used because it contains abundant natural matrix components such as laminin and collagen, which provide a suitable environment for cell adhesion, polarization, and survival. However, Matrigel has inherent limitations. Its batch-to-batch variability and undefined composition compromise experimental reproducibility and hinder quantitative analysis (Aisenbrey and Murphy, 2020). In this case, the hydrogel system with clear chemical composition is a good alternative. For example, the stiffness of polyethylene glycol (PEG) hydrogels can be tuned to mimic different matrix stiffness by precisely controlling the crosslinking density. These hydrogels can also be functionalized with cell adhesion motifs such as RGD peptides (Gandin et al., 2022). Recent studies have shown that PEG hydrogels with tunable stiffness support human pluripotent stem cell viability and morphogenesis (Indana et al., 2024; Seitz et al., 2024). Intermediate-stiffness PEG hydrogels (∼10 kPa elastic modulus) promote epiblast polarization and lumen formation (pro-amniotic cavity-like structures), whereas softer matrices (∼1 kPa) yield polarized but aberrant structures and stiffer matrices (≥40 kPa) inhibit lumenogenesis (Seitz et al., 2024). Stiffness, ligand type and density directly control stem cell fate (Gandin et al., 2022). Specific adhesion ligands are essential for establishing cell polarity and driving morphogenesis. In addition to conventional PEG, the incorporation of glycosaminoglycans into PEG-based hydrogels has emerged as a promising strategy to create biomimetic matrices that can sequester and present morphogens in a spatiotemporally controlled manner (Kühn et al., 2025).

To balance controllability and bioactivity, synthetic hydrogels are often integrated with biomaterials such as gelatin methacryloyl (GelMA) or decellularized extracellular matrix (dECM). On one hand, GelMA exhibits good cell adhesion and can tune its photo-curing mechanical properties (Chen et al., 2022). Its rate of degradation can be programmed by controlling the degree of methacrylation and aligning with the dynamic process of tissue growth (Shirahama et al., 2016). In stem cell culture, the tunable mechanical properties of GelMA hydrogels have been exploited to create supportive microenvironments that facilitate controlled morphogenesis and tissue remodeling (Clerkin et al., 2025; Luo et al., 2025). Unlike GelMA, tissue-specific dECM hydrogels retain more of the complex biochemical signals of the *in vivo* microenvironment (Giobbe et al., 2019; Sart et al., 2014). They may therefore better guide the formation of target organ-like structures such as liver buds and intestinal tubes (Giobbe et al., 2019; Abilez et al., 2025). dECM hydrogels derived from embryonic tissues have been shown to preserve native biochemical signals and support lineage specification and self-organization in early developmental models (Sart et al., 2014). The hydrogel network formed by dECM is inherently functionalized with native biological macromolecules to mimic the extracellular environment. Porosity of above two kinds of materials will influence the diffusion efficiency of nutrient inside the hydrogel, while it also decides whether the cell aggregate can undergo further three-dimensional rearrangement and migration. It is a physical factor which determines the transformation process from simple cell cluster to complex cavity structure. In addition to these biopolymer based systems, alginate hydrogels have been explored as alternative matrices for embryogenesis research. Their ultra-low protein adsorption and tunable mechanical properties allow researchers to decouple biochemical and biophysical signals. Recent studies have used alginate hydrogels to investigate how matrix stiffness and viscoelasticity affect early lineage specification in stem cell based developmental models (Indana et al., 2024).

The emergence of dynamic culture systems alongside static culture represents a transition from culture to biomanufacturing. The rotating bioreactor prevents cell aggregates sedimentation by gentle mixing. It also promotes more uniform transport of nutrients and oxygen, which supports the survival and growth of larger organoids (DiStefano et al., 2018). The perfusion bioreactor system can mimic the microfluidic environment that exists in the early stage of embryo development *in vivo* (Korin et al., 2009). Fluid shear force serves two functions. It provides a mechanical stimulus that induces cell polarization and differentiation (Tharp and Weaver, 2018; Babaliari et al., 2025). It also helps maintain stable morphogen concentration gradients, which are required for patterning body axes such as the anterior-posterior axis (Nonaka et al., 1998; Huljev et al., 2023). The combination of engineered hydrogel scaffolds and perfusion systems enables precise control over morphogen gradients and mechanical signals, thereby improving the reproducibility of embryo model formation and tissue architecture (Sun et al., 2023). The engineering strategies discussed above, which include microwell-based confinement, synthetic hydrogels, microfluidic gradient control, and bioreactor systems, are not limited to blastoid or gastruloid generation. These approaches have also been successfully applied to other PSC-based embryo models. Examples include PASE, bilaminoids, and SEM models. This demonstrates their general utility in guiding self-organization and morphoenesis across diverse model systems. To facilitate cross-method comparison and provide a quick reference for readers, we summarize the key features, advantages, and limitations of these engineering strategies in Table 1.

**TABLE 1:** Comparison of key engineering strategies for embryo model construction.

Engineering strategy	Key features	Advantages	Limitations	Applications in embryo models
Microwells (Chen et al., 2023; Iworima et al., 2023)	Physical confinement; standardize aggregate size and geometry	High throughput; reproducible aggregate formation; scalable	Lack dynamic control; physical confinement may limit self-organization	Blastoids, gastruloids
Synthetic hydrogels (Indana et al., 2024; Seitz et al., 2024; Limasale et al., 2025)	Chemically defined composition; tunable stiffness, ligand density, and degradation kinetics	High reproducibility; defined physicochemical cues; replace Matrigel	May lack native complexity; require careful optimization	Epi models (lumenogenesis)
Bioreactors (rotating/perfusion) (DiStefano et al., 2018)	Dynamic culture; enhanced nutrient and oxygen transport; fluid shear stress	Support prolonged culture; improve homogeneity; scale-up capability	Equipment intensive; shear stress may affect sensitive structures	Gastruloids, organoids
Microfluidics (Sun et al., 2023; Zheng et al., 2021)	Precise morphogen gradients; spatiotemporal control of signaling cues	User-defined concentration profiles; high spatial precision	Require specialized fabrication; limited throughput	Gastruloids, PASE
Optogenetics (Legnini et al., 2023)	Light-controlled pathway activation; high spatiotemporal resolution	Non-invasive; on-demand activation/inhibition	Require transgenic lines; potential phototoxicity	Emerging applications in embryo models

Currently, engineered systems based on static culture conditions are well developed. However, technologies such as rotating bioreactors remain underutilized for improving nutrient homogeneity and providing mechanical stimulation (DiStefano et al., 2018). Micropatterning approaches that spatially confine cell adhesion are underdeveloped. These methods can help researchers understand how physical confinement on a patterned surface guides cells to break their initial symmetry and acquire distinct fates. Future integration of rotating bioreactors and micropatterning, along with embryo-endometrium co-culture systems, will enable more precise control over self-organization through uniform physical forces and defined cellular topology.

### 3.3 Spatiotemporal manipulation of signaling pathways

Fine temporal control is required to program development in embryo models (Figure 5). Critical developmental signals are generally transitory rather than persistently active, such as the WNT-Nodal signaling relay establishing axiality. This represents precise biochemical control of developmental processes. Transient induction of primitive streak-like structures by brief CHIR99021 treatment can trigger endogenous Nodal signaling waves. In contrast, persistent WNT activation leads to improper patterning (Kempf et al., 2016; Mc et al., 2024). This suggests that subsequent Nodal signaling waves act as the direct drivers of mesendoderm specification and axial extension. Successful development also depends on the precise timing of signal transitions. In addition to temporal control, spatial control is equally critical for coordinating cell fate decisions during embryogenesis.

**FIGURE 5:**
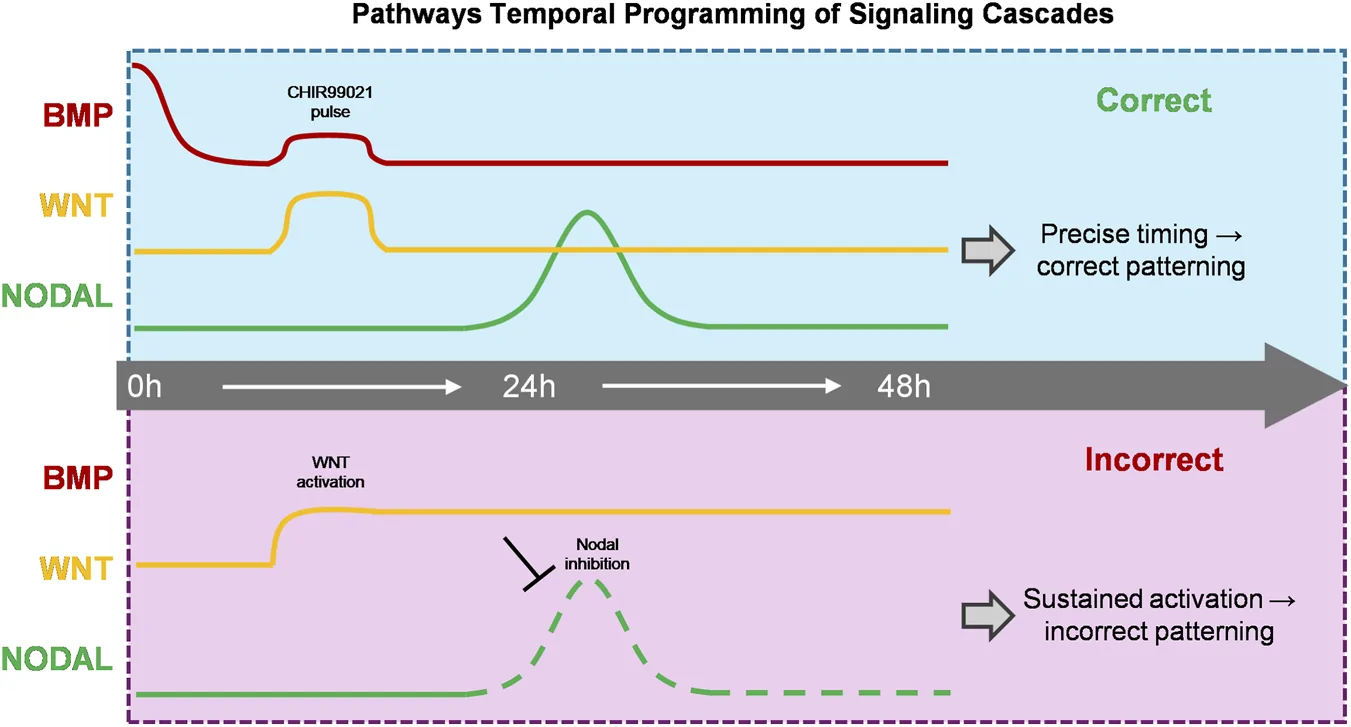
Hierarchical and complementary relationships of core signaling pathways guiding embryo model formation.

The schematic illustrates the hierarchical and complementary relationships of BMP, WNT, and NODAL pathways guiding embryo self-organization. The temporal activation cascade (BMP → WNT → NODAL) is depicted across 48 h. Correct patterning requires precise timing: a transient CHIR99021 pulse (WNT agonist) induces BRA/TBXT and triggers axial elongation, followed by NODAL-driven mesendoderm specification. Sustained WNT activation causes improper patterning. NODAL/Activin inhibition by SB431542 blocks mesendoderm and impairs axial elongation. These pathways function as an integrated network: BMP drives extraembryonic fates peripherally, while WNT and NODAL enrich posteriorly to pattern the embryonic axis. Abbreviations: BMP, bone morphogenetic protein; WNT, wingless-related integration site; NODAL, Nodal growth differentiation factor; h, hour.

In addition to temporal control, spatial control is equally critical for coordinating cell fate decisions during embryogenesis. In systems such as micropatterned cultures, gradients of established or inducible signals may be established by cells themselves through properties of local prepattern (Tewary et al., 2019). Studies have shown that spontaneous apical BMP4 stimulation and cell density dependent lateral localization of TGF-β receptors may establish a prepattern for differential signal response (Jo et al., 2022). This strategy recapitulates the BMP4-Noggin antagonism that patterns the dorsal-ventral axis. High BMP activity promotes ventral ectoderm (epidermis), while Noggin-mediated BMP inhibition allows dorsal ectoderm to acquire a SOX2^+^ identity. In the context of micropatterned gastruloids, this gradient drives differentiation from peripheral BMP-high regions to central BMP-low regions, analogous to the transition from ventral to dorsal ectoderm.

Beyond cell-autonomous gradient formation, engineered approaches have been developed to actively control morphogen gradients. Microfluidic devices offer a powerful platform for establishing stable, user-defined morphogen concentration profiles in 3D cultures. For example, Zheng et al. developed a passive diffusion microfluidic system that enables controlled delivery of BMP signaling to human pluripotent stem cell-derived post-implantation embryo models, driving symmetry breaking and amnion-epiblast patterning (Zheng et al., 2021). This approach demonstrates that precise spatiotemporal control of morphogen delivery is achievable and beneficial for modeling early human development.

In addition to microfluidics, materials themselves can serve as gradient controllers. Heparin-functionalized hydrogels, for example, can bind and release morphogens in a controlled manner to establish spatial gradients (Limasale et al., 2020). Recent advances in microgel technologies have further enhanced gradient resolution to the cellular level. Heparin-based microgels have been developed to present morphogens with high spatial precision (Kühn et al., 2025), and DNA-based microgels offer an alternative approach for generating programmable morphogen gradients (Afting et al., 2024). These material-based strategies complement microfluidic approaches by providing stable, local sources of morphogen signaling within 3D culture environments.

Efficient formation of blastocyst models is dependent on intervention in certain signaling pathways at defined time points. The core approach to forming blastocyst models involves inhibiting multiple signaling pathways with PALLY medium (PD0325901, A83-01, LPA, hLIF, and Y-27632) added between the 24- to 48-h period after cell aggregation (Kagawa et al., 2022; Heidari Khoei et al., 2023). This combination suppresses the MEK/ERK pathway (PD0325901), TGF-β/NODAL signaling (A83-01), activates Hippo-YAP via LPA receptor, maintains pluripotency through hLIF, and reduces cell death with ROCK inhibition (Y-27632), collectively promoting trophoblast specification and ICM self-assembly. This multi-pathway inhibition enables efficient specification of trophoblasts and facilitates self-assembly of ICM lineages. Among these inhibitors, LPA-induced Hippo pathway inhibition is especially important when formation efficiencies above 70% are required. In contrast, formation of gastrulation models focuses on harnessing the cell’s own self-organizing ability. There is a standard protocol for generating various types of gastrulation models, which consists of simply secreting BMP4 uniformly onto the culture medium for 42 h after plating cells on a micropatterned surface (E et al., 2016). This standard protocol combines three elements: apical application of BMP4, cell density-dependent receptor localization, and BMP4-induced Noggin expression. Together, these elements create a local activation–long-range inhibition system. This system generates a self-organizing signaling gradient from the colony edge to the center.

For more sophisticated spatial patterning models, many state-of-the-art works have adopted engineered approaches to actively assemble signaling gradients of interest. These strategies include three approaches (Fan et al., 2022; Sun et al., 2023; Legnini et al., 2023). First, morphogen-functionalized hydrogels serve as immobilized local signal sources. Second, microfluidic devices maintain stable, user-defined morphogen concentration profiles in 3D cultures via controlled convective and diffusive mass transfer. Third, optogenetic tools enable non-invasive, on-demand activation or inhibition of target pathways with high spatiotemporal precision using light. These technologies hold potent abilities to mimic dynamic patterns of endogenous embryonic signaling and therefore more tightly control guidance of cell self-organization and progressively achieve the goal of programming development in a dish.

Beyond these static and microfluidic systems, dynamic culture platforms have emerged as a critical advancement for achieving high-fidelity embryogenesis. Standard rotating bioreactors have been used to improve nutrient homogeneity, but controlled rolling culture platforms represent a more recent breakthrough. Tarazi et al. demonstrated that mouse embryo models generated from naïve ESCs can develop through gastrulation to early organogenesis when cultured in a controlled rolling platform (Tarazi et al., 2022). This approach provides fluid shear and dynamic mechanical cues that more closely mimic the *in vivo* environment, enabling developmental progression that static culture cannot support. Weatherbee et al. subsequently extended this rolling culture system to human embryo models, achieving post-implantation development up to early organogenesis (Weatherbee et al., 2023). These studies collectively demonstrate that dynamic culture platforms are not merely an incremental improvement but are essential for achieving high-fidelity developmental progression in embryo models. The integration of engineered hydrogel scaffolds with perfusion systems further enables precise control over morphogen gradients and mechanical signals, thereby improving the reproducibility of embryo model formation and tissue architecture.

## 4 Embryonic models: from blastoids to gastruloids

### 4.1 Blastoids: modeling the pre-implantation stage

Using the concepts and technical approaches described above, researchers have generated a series of embryo models that can capture development from the blastocyst stage through early organogenesis.

We start with the pre-implantation stage. Models at this stage seek to recapitulate the human blastocyst at 5–7 days after fertilization. The successful derivation of blastoids, complete structures containing the ICM, TE, and PrE is a remarkable achievement (Kagawa et al., 2022; Liu et al., 2021). Therefore, blastoids represent an exciting new tool to study the early events of human embryonic development.

Blastoids are synthetically assembled based on our knowledge of developmental biology. This involves 3D induction of PSCs that are pre-specified into different developmental states. Regardless of whether one aggregates PSCs directly or cultures naïve PSCs in 3D with specific cytokines (e.g., LIF, BMP4), the core idea is to take advantage of the inbuilt capability of these cells to give rise to both embryonic and extraembryonic lineages (Dong et al., 2020; Yanagida et al., 2021; Wei et al., 2024). This approach applies the preceding developmental principles: modulating Hippo regulators mimics peripheral polarity loss to drive trophoblast-like differentiation, while controlling WNT and Nodal signaling segregates Epi and PrE lineages (Jiao et al., 2025; Linneberg-Agerholm et al., 2019).

High-quality blastoids display remarkable morphological and molecular similarity to natural blastocysts. Morphologically, they form a separate, fluid-filled blastocoel-like cavity, surrounded by a monolayer of epithelial-like trophoblast cells and an inner cell mass-like cluster. Single-cell RNA sequencing analysis has further shown that these structures recapitulate the transcriptomic signature of pre-implantation embryos (Balubaid et al., 2024). They strongly express Epi markers (e.g., OCT4, NANOG), PrE markers (e.g., GATA6, SOX17) and trophoblast markers (e.g., CDX2, GATA3) (Balubaid et al., 2024; Kagawa et al., 2022; Fan et al., 2021). More impressively, several recent studies have shown that human blastoids can attach to endometrial organoids *in vitro* and induce a decidualization response, modeling the initial phase of implantation (Heidari Khoei et al., 2023).

The generation of blastoids represents a significant advance in developmental biology. These models allow researchers to track cell fate transitions from totipotency or pluripotency to differentiation. By using live imaging and tracking individual cells over time, one can follow the process of a cell making a fate choice. This system has confirmed that a cell’s position (inside vs. outside) within the blastocyst is directly correlated with its subsequent fate (Zhang et al., 2023). Additionally, by connecting disease modeling technologies to blastoid systems, one can directly test the impact of genetic defects that lead to early pregnancy failure in a human context, thus discerning the genetic basis of early developmental arrest (Dong et al., 2025).

Blastoids hold much promise for development and research, but they also have limitations. Developmental efficiency can vary greatly, with many models lacking the proper cell type proportions, degree of structural accuracy, and developmental synchrony with natural embryos. More critically, the great majority of human blastoids have extremely limited post-implantation potential and do not yield authentic embryonic tissues (Liu K. et al., 2023). As such, blastoids are currently most useful for studying pre-implantation development.

### 4.2 Gastruloids: modeling the post-implantation stage

After the successful modeling of the pre-implantation blastocyst, a major effort has been placed in generating embryo models that can recapitulate the early post-implantation stages (i.e., days 7–14 of human development) where morphogenetic events such as Epi polarization, formation of the amniotic and yolk sac cavities, and initial establishment of the primary body axis take place. While studying natural human embryos during this stage presents technical and ethical challenges, embryo models offer a viable and ethically acceptable alternative for investigating this developmental “black box”.

Moris et al. established the first human gastruloid model (Moris et al., 2020). They showed that aggregates of hESCs can undergo axial elongation and organize along an anteroposterior axis. This process recapitulates key aspects of early post-implantation development without extraembryonic tissues. Due to the increased cellular diversity, germ layer complexity, and highly organized architecture of post-implantation embryos, the methods used for the construction of gastruloid models are also more complex (Steventon et al., 2021). Two main approaches are currently employed to generate gastruloids. The first consists of extended culture of blastoids where extended *in vitro* culture conditions are used to mimic implantation (Karvas et al., 2023). The second involves direct aggregation of primed PSCs or EPSCs in 3D culture, followed by temporal modulation of signaling pathways (e.g., WNT, BMP, and NODAL) to induce symmetry breaking and axial elongation (Liu L. et al., 2023; Moris et al., 2020). Both strategies directly translate the engineering principles outlined in Section 3, combining biochemical cues and the physical microenvironment to guide gastruloid morphogenesis collectively.

Using human extended pluripotent stem cells (hEPSCs), peri-gastruloids were generated that can recapitulate steps of development up to early organogenesis (Liu L. et al., 2023). These models can recapitulate amniotic and yolk sac like cavity formation, bilaminar and trilaminar disc formation, primitive streak like patterning, gastrulation movements, and primordial germ cell like cells specification. Expression of key lineage markers was assessed by transcriptomics: SOX2, OCT4, and NANOG in the Epi; SOX17, GATA4, and GATA6 in the hypoblast; T in the primitive streak and mesoderm. Single-cell RNA sequencing also unveiled transcriptional similarities with human as well as non-human primate embryos.

Despite these achievements, gastruloids still have important limitations (Liu L. et al., 2023). First, gastruloids lack functional trophoblast cells and therefore cannot be used to model chorionic cavity formation or embryo-uterine crosstalk. Second, developmental progression tends to stall around 11–13 days in culture and the spatial organization of the amnion, Epi and yolk sac is sometimes compromised. Finally, variability in differentiation efficiency across hEPSC lines remains an issue.

Beyond classical gastruloids, models have been developed to study axial patterning and organ-specific development. For example, human gastruloid organoids treated with CHIR99021 display posterior expression of BRA (TBXT) and CDX2, as well as anterior expression of SOX2 and GATA6, with posterior regions also showing expression of LFNG, MESP2, and other somitogenesis-related genes and HOX family members. Spatial transcriptomic profiling of an intact Carnegie stage 9 human embryo has recently provided a high-resolution reference atlas of early organogenesis (Li et al., 2026), against which stem-cell-derived models can be benchmarked. Nevertheless, these stem-cell-derived gastruloids lack extraembryonic tissues and exhibit limited self-organization. To provide a systematic overview of the key differences between blastoid and gastruloid models, we summarize their starting cells, culture conditions, developmental stages, lineages, markers, efficiency, and limitations in Table 2.

**TABLE 2:** Comparison of blastoid and gastruloid models. The key features, capabilities, and limitations of the two major classes of embryo models are summarized to facilitate cross-model comparison.

Model type	Blastoids	Gastruloids/Peri-gastruloids
Starting cells	Naïve PSCs (hESCs/hiPSCs)	Primed PSCs (hESCs/hiPSCs) or EPSCs
Culture conditions	3D aggregation + PALLY medium	Micropatterned culture + BMP4/CHIR99021
Developmental stage modeled	Pre-implantation (blastocyst, ∼Day 5–7)	Post-implantation (gastrulation, ∼Day 7–14)
Lineages present	TE, ICM (Epi + PrE)	Epi, mesoderm, endoderm, (limited extraembryonic)
Key markers	TE: CDX2, GATA3; Epi: OCT4, NANOG; PrE: GATA6, SOX17	Epi: SOX2/OCT4; Mesoderm: BRA/T; Endoderm: SOX17
Key morphogenetic events	Blastocoel cavity formation, lineage segregation	Symmetry breaking, axial elongation, germ layer specification
Efficiency	≥70% (with LPA/Hippo inhibition)	Variable, line-dependent
Limitations	Limited post-implantation potential, no functional extraembryonic tissues	Lack vascularization, no trophoblast, developmental arrest ∼ Day 11–13
Key references	Kagawa et al. (2022); Liu et al. (2021)	Sozen et al. (2018); Liu et al. (2023a)

Beyond blastoids and gastruloids, several other PSC-based models have been developed to recapitulate specific aspects of human embryogenesis. The bilaminoid model, generated by co-culture of GATA6-induced hypoblast-like cells with naïve PSCs, forms a bilaminar disc-like structure that recapitulates epiblast-hypoblast interactions and primitive streak formation (Guo et al., 2024). The SEM (stem cell-based embryo model) assembles multiple stem cell types to generate structures resembling the amnion, yolk sac, and primitive streak (Gantner et al., 2025). The eX-embryoid, formed by co-culture of primed hiPSCs with GATA6-expressing cells, models yolk sac hematopoiesis and extraembryonic niche formation (Weatherbee et al., 2023). The post-implantation amniotic sac embryoid (PASE) recapitulates amnion development and Epi polarization during early post-implantation stages (Shao et al., 2017; Zhen et al., 2019). These models complement blastoids and gastruloids by providing platforms to study specific developmental processes that are not captured by the more generalized models.

While blastoids and gastruloids have successfully recapitulated many aspects of early embryonic development, they lack vascular components. This limitation prevents them from modeling the interactions between developing tissues and the circulatory system, which are essential for later organogenesis. Vascularized cardiac-vascular organoids (cVOs) have emerged as a complementary system to study how vascular networks form and integrate with developing tissues. Progress has also been made towards developing vascularized cVOs, which are aggregates containing cardiomyocytes, endothelial cells, smooth muscle cells, pericytes and epicardial cells that form branched vascular networks (Abilez et al., 2025). These cVOs share transcriptional and cellular features with early human fetal hearts, including activation of NOTCH, BMP and VEGF signaling. However, as aggregates, they still show important functional and structural immaturity, such as low proportions of cardiomyocytes and endothelial cells, lack of hematopoietic cells, and immature electrophysiological properties.

In summary, gastruloids and related organoid models have greatly improved our ability to study post-implantation human development *in vitro*. Gastruloids successfully recapitulate aspects of axial patterning, germ layer specification and organogenesis. However, limitations such as the lack of extraembryonic lineages, limited developmental potential, and structural or functional immaturity highlight the need for further development. Future strategies aimed at incorporating extraembryonic lineages, improving structural fidelity, and extending developmental competence will be important to more fully model human embryogenesis.

## 5 Discussion

### 5.1 Translational applications and biomanufacturing challenges

Beyond their value for developmental biology, embryo-like models have significant potential for translational medicine and industrial applications due to their engineered nature. However, transitioning these models from laboratory settings to large-scale, reproducible applications faces several engineering challenges.

In drug discovery and toxicity screening, human embryo-like models offer a species-specific, scalable *in vitro* platform for evaluating compound effects on early development. Compared with traditional animal models, they more directly reveal human-specific developmental toxicity pathways and hold promise for replacing certain animal experiments in developmental toxicity testing. For example, blastoids can model drug-induced interference with embryo implantation, providing a physiologically relevant platform to study the molecular mechanisms of implantation failure and to screen compounds that may affect this critical process. A recent study established a 3D on-chip implantation model using human blastoids co-cultured with a bioengineered endometrial tissue called an endometrioid (Li et al., 2026). This system successfully recapitulated key events of human implantation and early post-implantation development. Importantly, when modeling implantation using samples from patients with recurrent implantation failure (RIF), the researchers observed significantly reduced blastoid implantation capability. Furthermore, a targeted screen of FDA-approved compounds identified candidates that markedly enhanced implantation efficiency in RIF-derived endometrioids. This demonstrates the platform’s utility for identifying therapeutic strategies to improve embryo-endometrium interaction. Gastruloids and organ-specific models, in contrast, enable screening for compounds that cause congenital malformations of the heart or other organ systems, facilitating earlier risk identification in drug development (Kagawa et al., 2022). Gastruloids have also been developed as a practical platform for teratogenicity testing. A proof-of-concept study demonstrated that both mouse and human gastruloids can be used to quantitatively assess the effects of teratogenic compounds (Mantziou et al., 2021; Amel et al., 2023). Morphological readouts, such as reduced axial elongation and decreased size, combined with lineage-specific reporter expression (e.g., SOX2 for ectoderm, BRA for mesoderm, SOX17 for endoderm), allowed the detection of multi-lineage differentiation defects and disrupted axial patterning. Significantly, the system recapitulated known species-specific susceptibilities, such as the human-specific effect of thalidomide, highlighting its potential as a more predictive alternative to animal-based developmental toxicity assays.

In disease modeling, combining iPSC technology with embryo-like models has created a new strategy for investigating congenital disorders. By using iPSCs derived from patients with specific genetic diseases to generate embryo-like models, researchers can recapitulate abnormal developmental processes *in vitro*. This approach provides a dynamic, experimentally accessible human system for studying the cellular and molecular mechanisms of complex diseases such as congenital heart disease and spina bifida, while also offering a platform for personalized medicine research (Mashali et al., 2025).

Heterogeneity in initial cell conditions contributes to variability in blastoid formation efficiency (Section 4.1) and in gastruloid differentiation across cell lines (Section 4.2). To address this, dynamic culture platforms provide more consistent mechanical and biochemical environments than static culture. For example, the controlled rolling systems used by Tarazi et al. and Weatherbee et al. have been shown to improve reproducibility across experiments (Tarazi et al., 2022; Weatherbee et al., 2023). Automation can standardize critical steps such as cell seeding density, the timing of small-molecule addition, and embryoid aggregation size (Lattin et al., 2024; Lee et al., 2024). By minimizing pipetting errors and ensuring consistent aggregate formation, this approach directly reduces the operational variability that currently complicates cross-study comparisons. In addition, intelligent bioreactors equipped with sensors and feedback controls are needed to address the developmental arrest observed in current models around day 11–13 (Tarazi et al., 2022). To extend model survival beyond this barrier, bioreactors must monitor key physicochemical parameters: dissolved oxygen (to prevent hypoxia-induced differentiation), glucose and lactate concentrations (to assess metabolic shifts), and pH (to maintain stable buffering) (Lee et al., 2024). Real-time feedback control of these parameters could create a more stable microenvironment that more closely mimics *in vivo* conditions and supports prolonged development. Furthermore, replacing undefined Matrigel with chemically defined, tunable hydrogels can address the batch-to-batch variability that currently complicates quantitative comparisons between studies (Kim and Cha, 2025). Stiffness, RGD ligand density, and degradation kinetics must be precisely controlled to ensure consistent epithelial polarization and cavity formation across experiments. Overcoming these biomanufacturing challenges will enable embryo-like models to transition from nonstandard research tools into standardized biotechnology products for practical drug discovery and regenerative medicine applications.

### 5.2 Future perspectives

Although there have been notable advances, current embryo models show limited overlap with natural embryos and possess considerable gaps in terms of fidelity, completeness and complexity. Overcoming these limitations is a major challenge.

Most models differ from natural embryos in fundamental physical parameters. For example, they are generally much smaller, have fewer cells, and contain different cell type proportions. Blastoids have trophoblast lineages that, while capable of initiating attachment and decidualization responses *in vitro* (Li et al., 2026), remain functionally immature and lack the full trophoblast complexity required for modeling complete placentation (Rosner et al., 2025; Balubaid et al., 2024). Furthermore, *in vivo*, post-implantation development involves dynamic crosstalk with extraembryonic tissues such as the yolk sac and chorion, which provide nutrients and serve as signalling centres for primitive streak migration and organogenesis. Because models lack any functional extraembryonic components as well as the complex supporting structures required for maintaining development over time, they typically arrest around the time of primitive streak emergence.

Future models should strive to become more physiologically relevant by incorporating functional extraembryonic tissues. Two potential strategies exist. One is to establish co-culture conditions in which embryonic models are grown in close proximity to trophoblast or extraembryonic endoderm cells to recapitulate key tissue interactions critical during development. The other is to bioengineer novel 3D hydrogels that provide spatiotemporally controlled signalling inputs to drive the formation of extraembryonic mesoderm and structures such as the yolk sac. Beyond these biological strategies, the development of next-generation bioinks will also be important. These bioinks should provide supportive matrices for reproducible bioprinting of multi-tissue embryo assemblies. They should also possess tunable physicochemical properties. Future bioinks should have three key properties. They should enable programmable degradation kinetics for dynamic tissue remodeling. They should also allow controlled morphogen release to establish spatiotemporal signaling gradients. In addition, spatially graded stiffness is needed to guide mechanical cues during development.

The solutions discussed above must be integrated to overcome the current limitations of embryo models. Standardized cell preparation can reduce variability in starting cell populations. Defined hydrogel matrices can eliminate batch-to-batch variation in culture substrates. Intelligent bioreactor control can provide the stable physicochemical environment required for prolonged development. Together, these advances could generate models with higher fidelity and reproducibility than currently possible. As these models become more advanced, we must also establish adaptive ethical guidelines to ensure responsible use as they gain physiological relevance.

The ultimate goal is to generate a synthetic embryo model recapitulating all the major embryonic and extraembryonic compartments, including the Epi, mesoderm, hypoblast, amniotic cavity, yolk sac, and chorionic trophoblast. By combining stem cell biology, developmental genetics, and engineering tools, research on embryo-like models will transition from observational science to programmable developmental engineering. These collective advances will create a completely new experimental system to decode the principles of human life from its earliest stages in a dish.

## 6 Conclusion

Human embryo models have emerged as a platform that bridges stem cell biology, developmental biology, and bioengineering. This review has examined three core aspects of their construction: the selection and preparation of starting cell types, the design of engineered microenvironments, and the spatiotemporal control of signaling pathways. These include hESCs versus hiPSCs, primed versus naïve states, microwells, chemically defined hydrogels, dynamic bioreactors, the BMP-WNT-NODAL cascade, and morphogen gradients.

Despite notable progress, current models remain incomplete. They lack functional extraembryonic tissues, stall around primitive streak emergence, and show limited post-implantation potential. Reproducibility issues arising from cell line variability, matrix batch effects, and the stochastic nature of self-organization further limit their translational utility.

Addressing these limitations will require three advances: integrating trophoblast and extraembryonic endoderm lineages, developing next-generation bioinks with programmed physicochemical cues, and implementing closed-loop feedback control in bioreactor systems. Once these technical challenges are addressed, embryo models have the potential to move from proof-of-concept demonstrations to standardized platforms for drug screening, disease modeling, and regenerative medicine.
